# Additive effects of HLA alleles and innate immune genes determine viral outcome in HCV infection

**DOI:** 10.1136/gutjnl-2013-306287

**Published:** 2014-07-04

**Authors:** Karen Fitzmaurice, Jacob Hurst, Megan Dring, Andri Rauch, Paul J McLaren, Huldrych F Günthard, Clair Gardiner, Paul Klenerman

**Affiliations:** 1 Nuffield Department of Medicine, University of Oxford, Oxford, UK; 2 Institute of Emerging Infection, The Oxford Martin School, University of Oxford, Oxford, UK; 3 Natural Killer Cell Research Group, School of Biochemistry and Immunology, Trinity Biomedical Sciences Institute, Trinity College, Dublin 2, Ireland; 4 University Clinic of Infectious Diseases, University Hospital Bern and University of Bern, Bern, Switzerland; 5 Institute of Microbiology, University Hospital and University of Lausanne, Lausanne, Switzerland; 6 Division of Infectious Diseases and Hospital Epidemiology, University Hospital Zurich, University of Zurich, Zurich, Switzerland; 7 NIHR Biomedical Research Centre, John Radcliffe Hospital, Oxford, UK

**Keywords:** T Lymphocytes, Hepatitis C, HLA, Immunogenetics, Immunology in Hepatology

## Abstract

**Background:**

Chronic HCV infection is a leading cause of liver-related morbidity globally. The innate and adaptive immune responses are thought to be important in determining viral outcomes. Polymorphisms associated with the *IFNL3* (*IL28B*) gene are strongly associated with spontaneous clearance and treatment outcomes.

**Objective:**

This study investigates the importance of HLA genes in the context of genetic variation associated with the innate immune genes *IFNL3* and *KIR2DS3*.

**Design:**

We assess the collective influence of HLA and innate immune genes on viral outcomes in an Irish cohort of women (n=319) who had been infected from a single source as well as a more heterogeneous cohort (Swiss Cohort, n=461). In the Irish cohort, a number of HLA alleles are associated with different outcomes, and the impact of *IFNL3*-linked polymorphisms is profound.

**Results:**

Logistic regression was performed on data from the Irish cohort, and indicates that the *HLA*-A*03 (OR 0.36 (0.15 to 0.89), p=0.027) -B*27 (OR 0.12 (0.03 to 0.45), p=<0.001), -DRB1*01:01 (OR 0.2 (0.07 to 0.61), p=0.005), -DRB1*04:01 (OR 0.31 (0.12 to 0.85, p=0.02) and the CC *IFNL3* rs12979860 genotypes (OR 0.1 (0.04 to 0.23), p<0.001) are significantly associated with viral clearance. Furthermore, DQB1*02:01 (OR 4.2 (2.04 to 8.66), p=0.008), *KIR2DS3* (OR 4.36 (1.62 to 11.74), p=0.004) and the rs12979860 *IFNL3* ‘T’ allele are associated with chronic infection. This study finds no interactive effect between *IFNL3* and these Class I and II alleles in relation to viral clearance. There is a clear additive effect, however. Data from the Swiss cohort also confirms independent and additive effects of *HLA* Class I, II and *IFNL3* genes in their prediction of viral outcome.

**Conclusions:**

This data supports a critical role for the adaptive immune response in the control of HCV in concert with the innate immune response.

**Significance of this study**

**What is already known on this subject?**

- Host factors are considered to be an important component in the control of HCV infection.
- The HLA genes, as well as the innate immune genes *IFNL3* and *KIR*, are considered to be important determinants of viral outcomes, however, their interaction with each other has not been well studied.
- A single source outbreak of HCV provides us with a unique opportunity to understand the nature of these genetic influences in more detail as confounding factors are controlled.

**What are the new findings?**

- *HLA* Class I and II genes are significantly associated with viral outcomes even when the profound impact of *IFNL3* and *KIR2DS3* are considered.
- There is no evidence of a genetic interaction effect between the *HLA* Class I and II alleles and *IFNL3*.
- There is, however, a clear additive effect between the different genes indicating likely independent and separate but cumulative immune events.
- The results also indicate that there may be a differential effect with respect to *HLA* Class I alleles and viral outcomes according to the nature of the innate immune response.
- These data support a critical role for the adaptive immune response alongside the innate immune response in the control of HCV infection.

**How might it impact on clinical practice in the foreseeable future?**

- These data provide important insights to the immunopathogenesis of this illness which is of direct relevance to HCV vaccine studies and design.

## Introduction

Infection with HCV is one of the leading causes of liver-related mortality globally. Most persons who are exposed to HCV will develop persistent infection; however, a proportion (20–30%) spontaneously clear infection. Those patients with chronic HCV infection are at risk of developing liver cirrhosis and/or hepatocellular carcinoma.^1^ Much effort has aready been invested in understanding the factors that determine the differing outcomes in individuals but disentangling the impact of host and viral factors has been difficult. One of the reasons for this is that HCV exhibits substantial genetic heterogeneity as a consequence of its error-prone polymerase enzyme,^2^ ^3^ and high replication rates with virion production exceeding 10^12^ particles per day.^4^

Nonetheless, there are clear reports of host factors that are influential in determining viral outcome. The cellular immune response coordinated by CD4 and CD8 T cells appears to be important in this process. These responses are genetically predetermined by the nature of the host's major histocompatibility complex (MHC) molecules which dictate the antigen recognised in each individual. Certain *HLA* Class I and II genes have been linked to differing viral outcomes. Of the Class I genes, *HLA*-A*03, -B*27, -B*57 and C*01 have been shown to associate significantly with viral clearance.^5^ ^6^ Similarly, the Class II alleles have also been implicated—*HLA*-DRB1*01:01, 04:01 and DQB1*03:01 are associated with viral clearance, while others, such as DQB1*02:01 and DRB1*03:01, are associated with persistence.^5^ ^7^ Functional studies have linked the Class I associations (in the case of -A*03 and -B*27) to targeting of key protective CD8 T cell epitopes which appear to have a high fitness cost requiring the generation of multiple mutations.^8^ ^9^ The mechanism underpinning the protection associated with HLA Class II is not yet fully understood.

The innate immune response also appears to be crucial to viral containment. The interferon lambda 3 (*IFNL3)* gene encodes an innate interferon—IFN lambda 3 (IFNλ3) and a body of evidence supports a critical role for this cytokine in HCV infection. IFNλ3 is classified as a type III interferon. These cytokines possess antiviral and immunomodulatory activity mediated through the JAK-STAT pathway and can upregulate expression of interferon-stimulated genes (ISG).^10^ The importance of variants linked to this gene was first identified in a genome wide association study (GWAS) when a group of single nucleotide polymorphisms (SNP) identified at specific sites within a haplotype block were found to strongly correlate with either persistence or clearance of HCV in patients receiving interferon treatment.^11^ The exact mechanism responsible for this strong *IFNL3* effect remains unclear. However, despite the strong *IFNL3* effect, outcome is predicted in only about two-thirds of cases.^10^

As previously mentioned, much effort has been invested in determining the crucial host factors that help determine how HCV is controlled in humans. A number of important insights to this have arisen from the study of a cohort of Irish women who were accidentally exposed to HCV after receipt of doses of infected anti-D immunoglobulin.^12^ This cohort is defined by exposure to the same strain (genotype 1b) at a defined time point. The single-source outbreak of HCV infection presents an ideal study group in which to address the impact of host and viral factors, as host and viral heterogeneity are controlled for. The characteristics of this cohort have allowed several observations to be described, which have not been so clear-cut in other groups. Genetic studies have identified clear HLA Class I associations (table 1). A*03, B*27 and C*01 are associated with viral clearance whilst *HLA*-B*57 is not.^5^ Similarly, clear associations between *HLA* Class II genes and outcomes have been observed. *HLA*-DRB1*01:01, *04:01 and DRB1*15 have been linked to viral clearance, while DQB1*02:01 was associated with chronic infection.^5^ ^18^ Two recent studies also provide powerful evidence of the importance of *IFNL3* in determining outcomes in this group.^16^ ^17^ Dring *et al* have studied the impact of the rs12979860 *IFNL3*-linked polymorphism in more than 500 women derived from this cohort. They found the presence of the ‘T’ allele strongly predicted chronic infection (OR 7.38, p<10^−8^). They also found that the *KIR2DS3* gene in combination with the *HLA*-C2 (an epitope of *HLA*-C molecules that provides a ligand for some NK cell receptors) also predicted viral persistence (OR 1.90, p<0.002).^16^ Previous studies have also identified an effect of *KIR* genes on viral outcomes in HCV infection although not specifically *KIR2DS3.*^19–21^

**Table 1:** Genes previously associated with favourable and unfavourable outcomes within the Irish cohort

Protective	Deleterious	OR (95% CI)	References
*HLA*-B*27		7.99 (1.90 to 33.51)	5
*HLA*-A*03		2.43 (1.2 to 4.85)	5
	*HLA*-C*04	—	13
*HLA*-DRB1*01:01		4.71 (2.11 to 10.49)	5^,^ 14^,^ 15
DRB1*04:01		4.12 (2.04 to 8.34)	5
DRB1*15		2.2 (1.21 to 3.99)	5
	DQB1*02:01	0.27 (0.14 to 0.52)	5
	*KIR2DS3*	2.27 (1.28 to 4.02)	16
	*IL28B ‘T’*	7.59 (4.86 to 11.8)	16^,^ 17

In this study, we aimed to address a question, which has not previously been reported upon—to define the impact of genetic variation in innate immune genes (*IFNL3* and *KIR2DS3*) on the protective nature of MHC Class I and II, and were in a unique position to study this in the Irish cohort of women infected with a common inoculum of HCV from anti-D injections. We hypothesised that in patients who have a suboptimal innate immune response, a favourable adaptive immune response, such as that associated with protective *HLA* Class I and II alleles, might have a differential effect on viral outcomes or vice versa. As innate interferons may very well have an effect on the quality of the subsequent adaptive immune response, we also address whether *IFNL3* and the *HLA* Class I and II genes have separate, additive or interactive effects on HCV clearance.

## Methods

### Cohort

The study population is a group of Irish women who were exposed to HCV (genotype-1b) as a consequence of receipt of contaminated anti-D immunoglobulin in 1977. The characteristics of this cohort have been previously published.^12^ The subjects in this study were attending different centres in Ireland. Informed and written consent was obtained from each participant and ethical approval was obtained previously from the local centres. Our study group is comprised of 319 patients derived from the cohort (table 2) Complete *HLA* Class I, II, *KIR2DS3* and *IFNL3* genotyping was not available on all patients. In Group 1 (n=319), we had access to complete *HLA* Class I, *KIR2DS3* and *IFNL3* rs12979860 genotyping. Group 2 (n=213) is a subset of the first group: however, in this case, we also have full Class II typing.

**Table 2:** The Irish cohort has been divided into two study groups according to the availability of genotyping

	*IFNL3* T+	*IFNL*3 CC	*HLA* Class I	*HLA* Class II
Group 1 n=319	163	156	319	—
SR	29	94	124	—
Chronic	134	62	196	136
Group 2 n=213	110	103	213	213
SR	21	56	77	77
Chronic	89	47	136	136

SR, spontaneous resolver.

Data was also available from 461 patients derived from the Swiss HIV Cohort Study (previously described, and also in (http://www.shcs.ch).^17^ This is a multiple source cohort composed of HIV/HCV coinfected individuals (208 spontaneous clearers and 253 with chronic infection). These patients were infected with diverse HCV genotypes and were heterogeneous with respect to age, sex, ethnicity and viral co-infections (see online supplementary table S1). Data was available on *HLA* Class I, II and *IFNL3* rs4803217 genotypes. Written informed consent including for genetic testing had previously been obtained.

#### *IFNL3, KIR2DS3* and *HLA* genotyping

Genotyping for these variables (*IFNL3* SNP rs12979860, *KIR2DS3* and *HLA*) in the Irish cohort was performed previously and is described in previous publications.^5^ ^16^ ^18^ For the Swiss cohort, genotyping for the *IFNL3* linked polymorphism rs4803217 was performed. This SNP was chosen as a proxy (R^2^ of 0.92) for rs12979860 because the latter SNP was not genotyped in the frame of the Swiss cohort GWAS. HLA type was imputed using the SNP2HLA pipeline and a reference panel of 5225 individuals with MHC-wide dense SNP typing and 4-digit sequence-based HLA typing.^22^

### Statistical analysis

For the Irish data, Fisher's exact test and step-wise multivariate logistic regression analysis is used to assess the predictive contribution of *HLA* type in a model that also includes the patient's *IFNL3* status and *KIR2DS3* genotype. Stepwise logistic regression models were produced using forward and backward variable selection. The R step function was used to minimise Akaike Information Criteria (AIC). The additive effect of the selected *HLA* type, *IFNL3* and *KIR2DS3* was examined by hierarchical modelling and receiver operator curves (ROC). The interaction of *IFNL3* and the optimised *HLA* types was assessed within the regression models. For the Swiss data, univariate regression was performed for outcome for each HLA (see online supplementary data). The rest of the analysis follows the same procedures as the analysis for the Irish cohort. All analysis is carried out using R (http://www.r-project.org) V.3.10.

## Results

### *HLA* Class I alleles are associated with viral clearance

In this study, complete *HLA* Class I data was available on 319 patients derived from the Irish anti-D cohort (Group 1). A previous study has reported significant associations between Class I alleles and viral outcomes on a smaller subset of this cohort (n=247, table 1).^5^ We initially compared the frequencies of the Class I alleles in patients with chronic and resolved infection in these 319 patients. Our results also indicate a favourable association between *HLA*-A*03 (OR 0.5 (0.3 to 0.9), p=0.01) and B*27 (OR 0.3 (0.1 to 0.9), p=0.02) and viral clearance. Additionally, C*01 (OR 0.3 (0.08 to 1), p=0.03), C*12 (OR 0.4 (0.1 to 1), p=0.03) and B*15 (OR 0.3 (0.1 to 1), p=0.04) are significantly associated with viral clearance, while C*04 (OR 2 (1 to 4), p=0.04) is associated with persistent infection.

### *HLA*-A*03 is significantly protective only in the *IFNL3* ‘protective’ group, while B*27 and C*01 are protective only in the *IFNL3* ‘deleterious’ group

To address the effect of the *IFNL3* SNP rs12979860 on the *HLA* Class I associations, we then stratified the cohort according to carriage of the ‘protective’ CC genotype or ‘deleterious’ T+ genotype and re-analysed viral outcomes in these groups (table 3 and see online supplementary table S3 and S4). In those with the protective *IFNL3* genotype, *HLA*-A*03 is still strongly associated with viral clearance (OR 0.34 (0.14 to 0.8), p=0.007). The presence of *HLA*-A*03 is also more common in resolvers than in those with persistent infection in the deleterious *IFNL3* group (35% vs 25%, OR 0.6 (0.2 to 1.7), p=0.35) although this is no longer statistically significant. In the group stratified by the *IFNL3* ‘deleterious’ genotype, we identify significant associations between *HLA*-B*27, -C*01 and viral clearance (OR 0.12 (0.02 to 0.6), p=0.002 and OR 0.11 (0.02 to 0.6), p=0.005, respectively) and observe that this effect is not present in the ‘protective’ group (table 3). The number of patients who have these important alleles (B*27, C*01) is low, however.

**Table 3:** HLA Class I and II associations with viral outcome in patients from the Irish stratified by their *IFNL3* rs12979860 genotype (Fishers exact test)

*IFNL3* rs12979860 CC genotype					*IFNL3* rs12979860 T+ genotype			
*HLA*	Chronic n=62 (%)	Resolved n=94 (%)	OR (95% CI)	p Value	Chronic n=134 (%)	Resolved n=29 (%)	OR (95% CI)	p Value
A*03	10 (16)	34 (36)	0.34 (0.14 to 0.8)	0.007	33 (25)	10 (35)	0.6 (0.2 to 1.7)	0.35
B*27	4 (7)	8 (9)	0.7 (0.2 to 3)	0.77	4 (3)	6 (21)	0.12 (0.02 to 0.6)	0.002
C*01	2 (3)	5 (5)	0.6 (0.06 to 3.8)	0.7	3 (2)	5 (17)	0.11 (0.02 to 0.6)	0.005
B*15	3 (5)	7 (7)	0.6 (0.1 to 3)	0.74	3 (2)	4 (14)	0.2 (0.02 to 0.9)	0.02
Cw*16	11 (18)	6 (6)	3.1 (1 to 11)	0.035	7 (5)	7 (24)	0.3 (0.07 to 1.2)	0.04
C*09	5 (8)	1 (1)	8.1 (0.9 to 389)	0.037	4 (3)	1 (3)	0.9 (0.08 to 44)	1

**HLA**	**Chronic n=47**	**Resolved n=56**	**OR (95% CI)**	**p**	**Chronic n=89**	**Resolved n=21**	**OR (95% CI)**	**p**

DRB1*01:01	3 (5)	17 (18)	0.16 (0.03 to 0.6)	0.002	8 (6)	9 (31)	0.14 (0.04 to 0.5)	0.001
DQB1*05:01	8 (13)	20 (21)	0.4 (0.1 to 1)	0.045	11 (8)	9 (31)	0.2 (0.06 to 0.6)	0.003
DQB1*02:01	18 (29)	9 (10)	3.2 (1.2 to 9.3)	0.014	38 (28)	2 (7)	7 (1.5 to 66)	0.005
DRB1*03:01	17 (27)	9 (10)	2.9 (1.1 to 8.5)	0.024	38 (28)	2 (7)	7 (1.5 to 66)	0.005
DRB1*04:01	9 (15)	17 (18)	0.5 (0.2 to 1.5)	0.26	11 (8)	5 (17)	0.5 (0.1 to 2)	0.18
DQB1*02:02	14 (23)	4 (4)	5.4 (1.5 to 25)	0.004	19 (14)	4 (14)	1.2 (0.3 to 5.3)	1
DR*13:01	9	0	0	0	11	2	1.3 (0.3 to 13.4)	

*HLA* Class II data was available for 213 women (Group 2). Online supplementary table S5 shows the frequencies of the Class II alleles in patients with different outcomes in this analysis and are similar to previous studies.^5^ ^15^ ^18^ We also stratified the group according to the presence or absence of the beneficial *IFNL3* genotype. *HLA*-DQB1*02:01 and DRB1*03:01 were both significantly associated with chronic infection independently of the stratification. Similarly, DQB1*05:01 and DRB1*01:01 were both significantly enriched in those with viral clearance irrespective of the *IFNL3* genotype (table 3 and see online supplementary tables S6 and S7). These results suggest an independent effect of the Class II genes as a contributor to viral outcome.

### *HLA* Class I alleles predict viral outcome even when the impact of innate immune genes are considered

Logistic regression analysis was then used to generate an optimal multivariate model that would best predict viral outcome using the previously identified associations and genes noted to be in linkage disequilibrium. The *IFNL3* and *KIR2DS3* genotype were also incorporated. Table 4 presents the optimised regression model of class I *HLA*, *KIR2DS3* and *IFNL3* associations with viral clearance. Four of the *HLA*s are protective (A*03, B*15, C*01 and C*12) whilst C*09 is deleterious. These associations are maintained independently despite the presence of *IFNL3* within the regression even though *IFNL3* has a strong protective effect (OR 0.11, (0.06 to 0.19), p<0.001). The interactive effect of the *IFNL3-*associated polymorphisms with the Class I alleles was examined by including interaction term*s* in the logistic regression models. No significant interactions were detected. The additive effects were assessed by constructing hierarchical models. Models were constructed using only the optimal *HLA* I alleles or *KIR2DS3* or the *IFNL3* genotype and combinations of these predictors. As variables are added, there is a marked improvement in prediction of viral clearance. Online supplementary figure S2. displays the ROC generated by these models. The best model as defined by area under the curve (AUC 0.82) contains all the predictors. The *IFNL3* genotype (AUC 0.72) outperforms *KIR* (AUC 0.59) and *HLA* Class I alleles (AUC 0.65) when considered on their own.

**Table 4:** An optimised logistic regression model (incorporating *HLA* Class I, *IFNL3* and *KIR*) predicting viral clearance

Explanatory Variables	OR (95% CIs)	p Value
A*03	0.42 (0.23 to 0.76)	0.005
B*15	0.12 (0.03 to 0.52)	0.004
C*01	0.13 (0.03 to 0.5)	0.003
C*09	10.74 (1.42 to 81.3)	0.022
C*12	0.31 (0.11 to 0.86)	0.024
*IFNL3* CC versus CT/TT	0.11 (0.06 to 0.19)	<0.001
*KIR2DS3*	3.45 (1.77 to 6.72)	<0.001

The explanatory variables are selected by forward and reverse stepwise selection minimising Akaike Information Criteria. OR, CIs and p values are presented. The dataset consists of patients from the Irish cohort who have had their *HLA* Class I genotype determined (n=319).

*IFNL3* CC, protective genotype; *IFNL3* CT/TT, deleterious genotype.

### Predictive effect of immune genes are additive

We next generated a logistic regression model to predict viral outcome incorporating Class I and Class II alleles as well as *IFNL3* and *KIR2DS3* genotype. The regression included genes that were known to be enriched in patients with viral clearance or chronic infection, also genes noted to be in linkage disequilibrium with the former, in addition to the *IFNL3* and *KIR2DS3* genotype. *HLA*-A*03 (OR 0.36 (0.15 to 0.89), p=0.027), -B*27 (OR 0.12 (0.03 to 0.45), p=<0.001), *IFNL3* CC genotype (OR 0.1 (0.04 to 0.23), p<0.001), DRB1*01:01 (OR 0.2 (0.07 to 0.61), p=0.005), and DRB*04:01 (OR 0.31 (0.12 to 0.85, p=0.02) were significantly associated with viral clearance (table 5). DRB*13:01 (OR 5.95 (0.97 to 36.7), p=0.05), DQB1*02:01 (OR 4.2 (2.04 to 8.66), p=0.008), as well as the *IFNL3* ‘T’ allele and *KIR2DS3* (OR 4.36 (1.62 to 11.74), p=0.004) genotype were found to be associated with chronic infection (table 5.)

**Table 5:** An optimised logistic regression model (incorporating *HLA* Class I and II, *IFNL3* and *KIR*) predicting viral clearance

Explanatory variables	OR and confidence limits	p Value
A*02	2.32 (0.95 to 5.66)	0.064
A*03	0.36 (0.15 to 0.89)	0.027
A*11	2.36 (0.76 to 7.33)	0.137
B*07	0.49 (0.2 to 1.21)	0.122
B*27	0.12 (0.03 to 0.45)	<0.001
C*02	32.38 (1.62 to 645)	0.023
DR*04:01	0.31 (0.12 to 0.85)	0.022
DR*01:01	0.2 (0.07 to 0.61)	0.005
DR*13:01	5.95 (0.97 to 36.67)	0.054
DQ*02:01	4.2 (2.04 to 8.66)	0.008
*IFNL3* CC v CT/TT	0.1 (0.04 to 0.23)	<0.001
*KIR2DS3*	4.36 (1.62, 11.74)	0.004

The explanatory variables are selected with stepwise selection minimising Akaike Information Criteria (AIC). OR, CIs and p values are presented. The dataset consists of patients from the Irish cohort (n=213). *IFNL3* CC: protective genotype; *IFNL3* CT/TT: deleterious genotype.

As previously, we generated a model (ROC) to predict viral outcome incorporating the genes identified above (see online supplementary figure S3). This again demonstrates an additive effect of the variables. *HLA* Class I and II genes combined are able to predict viral outcome (AUC 0.82) better than either *KIR2DS3* (AUC 0.61) or *IFNL3* (AUC 0.69) alone. There is an improvement in prediction with the addition of all variables. The curve also demonstrates that the combined predictive HLA genes offer a good prediction of outcome (AUC 0.82) with only a slight improvement with the addition of *IFNL3* gene status and *KIR2DS3* (AUC 0.89).

### Additive effects of *HLA* and *IFNL3* genes are also present in an independent Swiss cohort

Given the unique characteristics of the Irish cohort as a group, we set out to address whether our observations in this dataset were reproducible in an independent heterogeneous population. *HLA* Class I and II as well as *IFNL3* genotypes (using the rs12979860 proxy SNP rs4803217, proxy R^2^=0.92) were available on 461 members of the Swiss cohort. The frequencies of the different *HLA* alleles in the Irish and Swiss cohorts are shown in online supplementary figures S4–S8. We again generated an optimised logistic regression model using the Swiss cohort genetic data to predict viral outcome (table 6). The strongest *HLA* predictor is *HLA*-A*11 which significantly predicts viral persistence in the Swiss cohort (p=0.004, OR 2.63 (CI 1.35 to 5.1). In the Irish cohort, there is also a trend towards an association between chronic infection and *HLA*-A*11 albeit not significant. Other studies have also suggested a predictive role for A*11 in other populations.^23^ The -DQB1*0301 allele is significantly associated with viral clearance (p=0.032, OR 0.66 (0.45 to 0.97)) as is the protective *IFNL3*-associated rs4803217-C allele.

**Table 6:** Optimised logistic regression model from the Swiss cohort (incorporating *HLA* Class I, II and *IFNL3*) predicting viral clearance

Explanatory variables	OR and confidence limits	p Value
A*11	2.63 (1.35 to 5.1)	0.004
A*23	3.14 (1.05 to 9.38)	0.041
A*29	1.92 (0.087 to 4.23)	0.096
B*08	0.62 (0.35 to 1.1)	0.1
B*49	0.44 (0.21 to 0.94)	0.034
C*14	0.37 (0.14 to 0.95)	0.033
DR*08:01	0.54 (0.24 to 1.19)	0.127
DQ*03:01	0.66 (0.45 to 0.97)	0.032
DQ*03:02	1.69 (0.97 to 2.96)	0.061
rs4803217-A (*IFNL3*)	2.43 (1.72 to 3.43)	<0.001

Explanatory variables are selected with stepwise selection minimising AIC. ORs, CIs and p values are presented. The dataset consists of patients from the Swiss cohort (n=461).

A ROC was generated to address the combined impact of the *HLA* alleles and *IFNL3* genotypes on viral outcomes in this cohort (see online supplementary figure S9). Similar to the results observed in the Irish cohort, we note an additive effect in the prediction of viral outcome between *HLA* alleles and *IFNL3* genotypes. This effect is less marked than that of the Irish cohort (0.65 AUC *IFNL3* alone ct. 0.72 for *HLA* and *IFNL3*). Nonetheless, it provides evidence that innate and adaptive immune events are independent and additive in the prediction of viral outcomes.

## Discussion

In this study, we aimed to define the established associations between MHC genes and viral outcomes in the context of the *IFNL3*-linked polymorphisms. We were in a unique position to address this, as we were able to investigate these relationships in a cohort of women who had been infected with HCV from a single source. We wished to address whether the observed MHC associations would remain significant in the context of the profound innate immune effect.

We first considered whether there might be a differential effect according to the characteristics of the innate immune response, by analysing the impact of carriage of either the protective or deleterious *IFNL3* genotype with respect to the *HLA* effect on viral outcomes. In our *HLA* Class I analysis, we did, to an extent, observe this effect. The presence of the protective alleles *HLA*-B*27 and –C*01 was significantly enriched in those with viral clearance if the unfavourable *IFNL3* genotype was present. In this cohort, the *HLA*-B*27 and C*01 alleles are infrequent (6% of population), and so the numbers in the study are limited. We also find that carriage of the protective *HLA*-A*03 allele was significantly associated with clearance only in the presence of the *IFNL3* CC genotype. These results indicate that there may indeed be a differential effect with respect to *HLA* Class I alleles and viral outcomes, according to the nature of the innate immune response. Larger studies would be required to confirm these observations however. In the case of Class II alleles, the results are more clear-cut suggesting an effect that is independent of the *IFNL3* genotype as the protective or deleterious effects of the Class II alleles were maintained irrespective of the stratification.

In our initial optimised multivariate model, we assessed the impact of *HLA* Class I alleles on viral outcomes in a regression incorporating the *IFNL3* status and *KIR2DS3* genotype. Results from this model reveal protective roles for the Class I alleles A*03, B*15, C*01 and C*12 and a deleterious role for C*09. We have previously studied the protective effect of *HLA*-A*03 and linked its effect to presentation of an immunodominant CD8 T cell epitope. *HLA*-B*15 has not previously been linked to protection in this cohort, but these results suggest a possible role. A large study by Hraber *et al* does report on an association between B*62 (a B*15 supertype) and viral clearance^24^ and functional studies also indicate the targeting of dominant CD8 T cell epitopes restricted by this allele.^25^ *HLA*-B*27 and -C*01 are inherited in strong linkage disequilibrium within this cohort. In our initial logistic regression analysis (n=319), C*01 appears as a significant predictor—however, in the later model which combines Class I and II alleles (n=213), it is the B*27 allele which is the best predictor of viral clearance. The numbers studied are low, however (n=22 for *HLA* B*27, Study group 1), and this makes it difficult to separate the effects of these closely linked genes. In this cohort, several other haplotypes are inherited in linkage disequilibrium.^5^ *HLA*-A*01-B*08-Cw*07 is linked to the Class II haplotype DRB1*03:01-DQB1*02:01—however, only DQB1*02:01 was noted to be a predictor of persistent infection in this study. The *HLA*-A*03 allele occurred in linkage with B*07-DRB1*15-DQB1*06:02. In this study, we find that only the A*03 component is predictive of viral clearance.

As well as being potential predictors in their own right as part of the adaptive immune response, *HLA* class I C alleles can be divided into two groups (C1 and C2) based on *KIR* ligand specificity. Prior work with this and other cohorts have considered the impact of this grouping, and found significant associations and interactions between viral clearance, *HLA* C1 or C2 and *IFNL3* genotype.^16^ ^21^ Suppiah *et al* have previously addressed the role of *HLA*-C alleles in combination with *IL28B* in predicting treatment outcomes in HCV. They found the *HLA*-C2C2 genotype to be over-represented in patients who failed treatment (OR 1.52), and that prediction of treatment failure improved from 66% to 80% if both genes were used. They noted that this effect was mainly additive, but there was some evidence that it was also partially due to genetic interaction.^21^ Dring *et al*^16^ report on a significant association between *KIR2DS3* on a *HLA* C2C2 background in the Irish cohort in predicting the development of persistent infection, and also report significant synergy between *KIR 2DS3* gene and the unfavourable *IFNL3* polymorphism in this cohort.

In this cohort, several *HLA* Class II genes have been firmly implicated as determinants of viral outcome (table 2).^5^ Other immunogenetic studies of HCV have identified different associations with Class II genes according to the population and genotype under investigation.^26^ Nonetheless, several *HLA* Class II genes appear to be important in diverse populations. A meta-analysis supported a strong association between DQB1*03:01 and DRB1*11:01 and viral clearance.^7^ Thio *et al*^27^ previously noted that the DRB1*03:01-DQB1*02:01 haplotype was also deleterious in Caucasian patients. In this study, we demonstrate that the Class II alleles (DRB1*01:01 and *04:01) retain their highly significant protective effect, while DQB1*02:01 retains its deleterious effect independently of *IFNL3* and *KIR2DS3*. Similar to our findings with the Class I alleles, we note that the prediction of viral outcomes are enhanced if these effects are combined, and that is not accounted for by a genetic interaction. These results support other recently published data. Duggal *et al* reported on a large GWAS of spontaneous resolution of HCV. This study combines data from different cohorts with 2401 persons included in the final analysis.^28^ Significant differences in allele frequencies between persons with spontaneous resolution and persistence were identified on chromosomes 19 and 6. On chromosome 19, the differences localised near *IFNL3* while on chromosome 6, differences localised near genes for *HLA* Class II and included a polymorphism near DQB1*03:01. In the Swiss cohort, we also noted a strong protective effect for this allele, and many other studies have observed this effect.^27–29^ The associations observed on chromosome 19 and 6 were noted to be independent and additive. In a smaller study, Mangia *et al* have assessed the impact of several protective Class II alleles on spontaneous viral outcomes in the context of the *IFNL3* SNP rs1297860 in a small cohort of thalassemic HCV-exposed patients. They have also demonstrated an independent and significant effect for the *HLA* Class II gene DQB1*03:01 in predicting viral clearance, and have shown that the effects of DQB1*03:01 and *IFNL3* are additive.^30^ In our study, and as previously reported, DQB1*03:01 was not found to be associated with viral outcomes. In the Irish population, DRB1*01:01 and DQB1*02:01 are strongly associated with clearance and chronic infection, respectively. The Class II allele DRB1*0401 is associated with clearance in the Irish cohort, while in the Swiss Cohort it is associated with persistence. These disparate results likely reflect the frequencies of different alleles within differing populations, and are likely to relate to the unique inoculum received by the Irish women and the related immune specificities. In this study, we note that the magnitude of prediction for the combined HLA I and II genetic data (AUC 0.82) is stronger than *IFNL3* alone (AUC 0.69) or *KIR2DS3* (0.61), which supports a critical role for the adaptive immune response in determining viral outcome.

A key question which arises when we study genetic factors in this unique population infected from a single source, is how do our findings extrapolate to more genetically heterogeneous populations infected with different genotypes. To address this, we were able to compare our findings with those from a separate European population. We demonstrate that *HLA* Class I and II alleles as well as *IFNL3* genes independently contribute to viral outcomes also. Furthermore, as we noted in our initial studies, we find that combining these different genotypes is additive in the prediction of viral outcome. This effect is less marked than noted in the Irish population, however, this is not a surprising result given the unique genetic and virological characteristics of this specific cohort. We observe different HLA associations in these cohorts which is most likely related to viral diversity (single strain vs diverse genotypes). Previous work on the Irish cohort has already illustrated the unique effects of a single infecting strain on viral evolution and immunodominance. In the Irish cohort, *HLA*-A*03 and -B*27 have been shown to mediate their protective effects through presentation of key immunodominant epitopes which have a high threshold to the development of immune escape variants. When these epitopes were examined in other diverse populations, these properties were not maintained.^31^ ^32^ The importance and influence of the source virus is illustrated in a study which investigated the roles of *HLA*-A*03, B*08 and B*27 in determining viral outcome in the East German single-source outbreak. By contrast with the Irish cohort, these alleles were neutral for disease outcome. Sequence analysis of the immunodominant epitopes revealed that pre-existing substitutions in the infection source of both cohorts influenced the impact of the corresponding *HLA*-allele. The epitopes presented by the ‘protective’ alleles *HLA*-A*03 and -B*27 in the Irish cohort contained substitutions in the source virus of the East German outbreak. These pre-existing substitutions altered selection pressure and viral evolution in the East German cohort.^33^

In conclusion, our results confirm the finding that *HLA* alleles are important predictors of viral response even given the powerful effect of *IFNL3-*linked polymorphisms. The observations demonstrated in the initial Irish studies have been reproducible in the Swiss cohort providing corroborative evidence that adaptive and innate immune events independently contribute to viral outcome, and that these events are additive.

## Supplementary Material

Web supplement
