# Supplementary material for: Additive effects of HLA alleles and innate immune genes determine viral outcome in HCV infection
Source: Gut. 2014 Jul 4;64(5):813–9. doi: 10.1136/gutjnl-2013-306287 (PMC4392199; doi:10.1136/gutjnl-2013-306287)
Supplement: Web supplement [file gutjnl-2013-306287-s1.pdf]

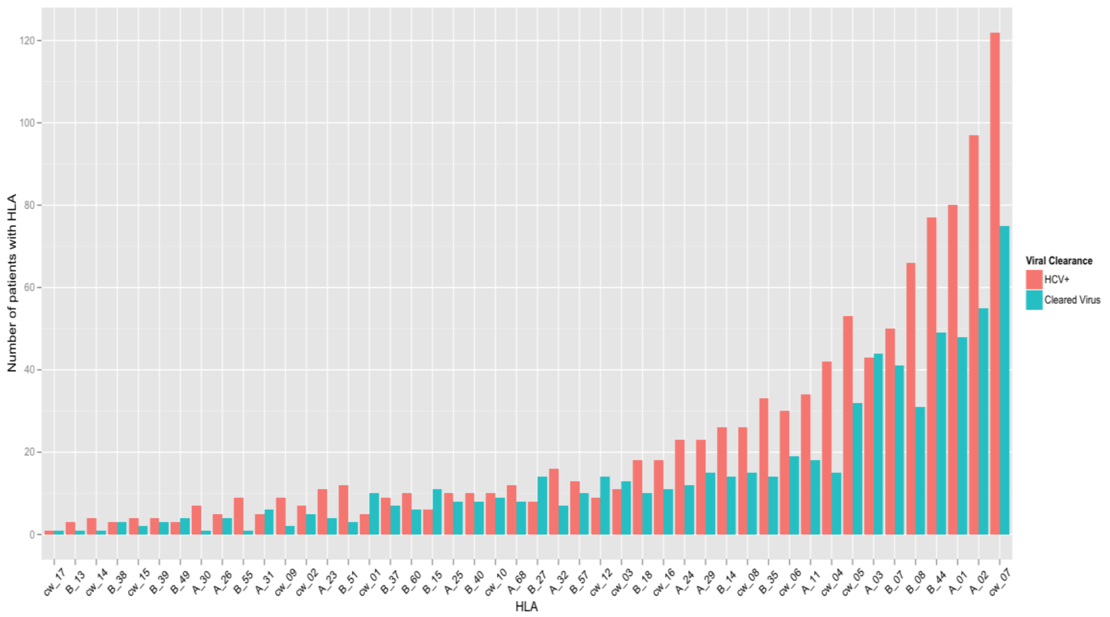


Supplementary Figure 1. HLA and viral clearance in the Irish Cohort. Number of patients with class I alleles within the Irish Cohort, the red bars indicate the number of patients who have not cleared the virus the blue bars indicate the number of patients who have cleared the virus.

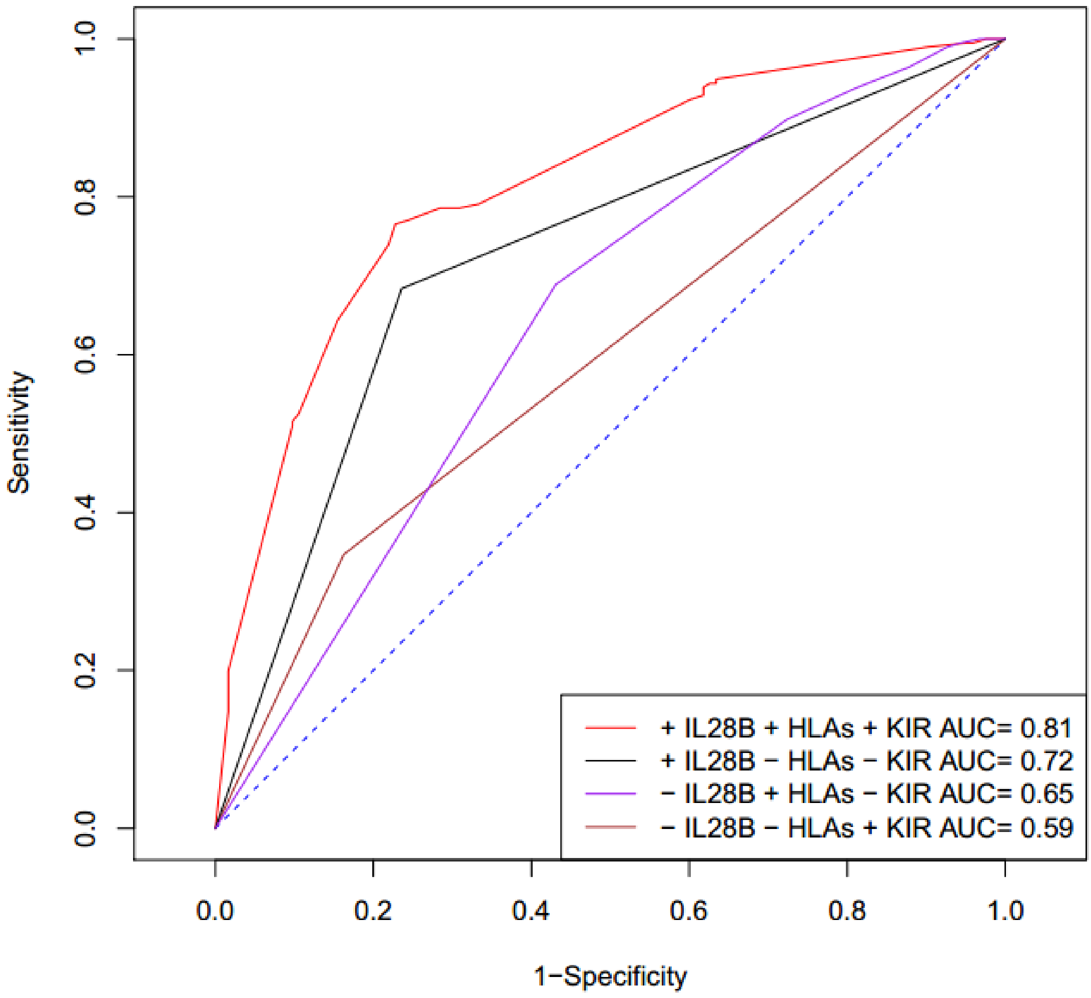


Supplementary Figure 2. ROC curve illustrating the additive performance of different regression models with and without the class I MHC, *IFNL3 (IL28B)* status and the *KIR2DS3* status within the Irish cohort.

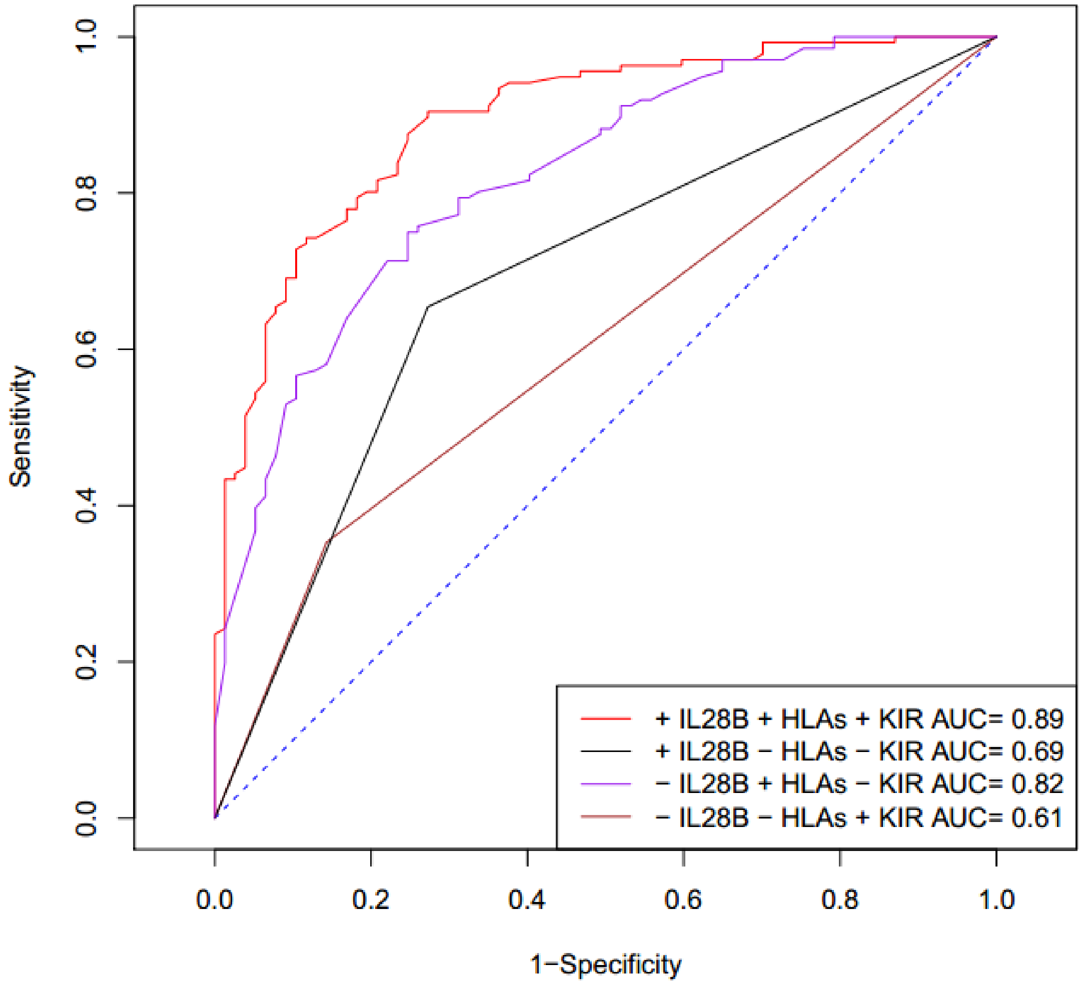


Supplementary Figure 3. ROC curve illustrating the performance of different regression models with and without the class I and class II MHC, *IFNL3* (*IL28B*) status and the *KIR2DS3* status within the Irish cohort.

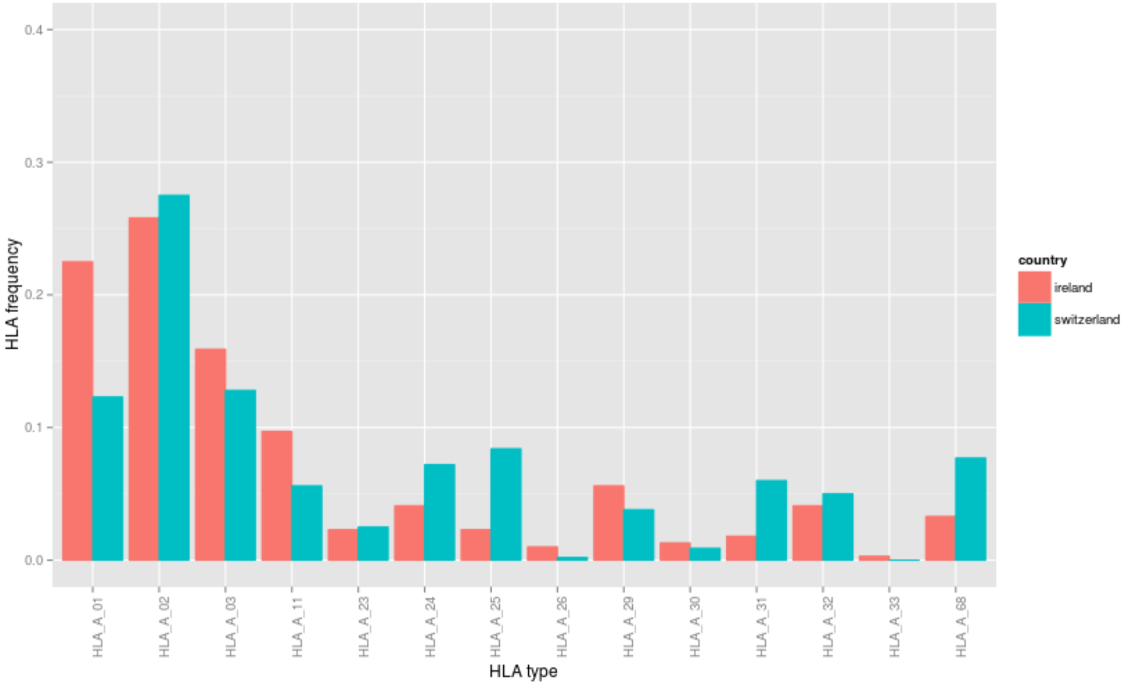


Supplementary Figure 4. Histogram of HLA-A diversity between the two populations.

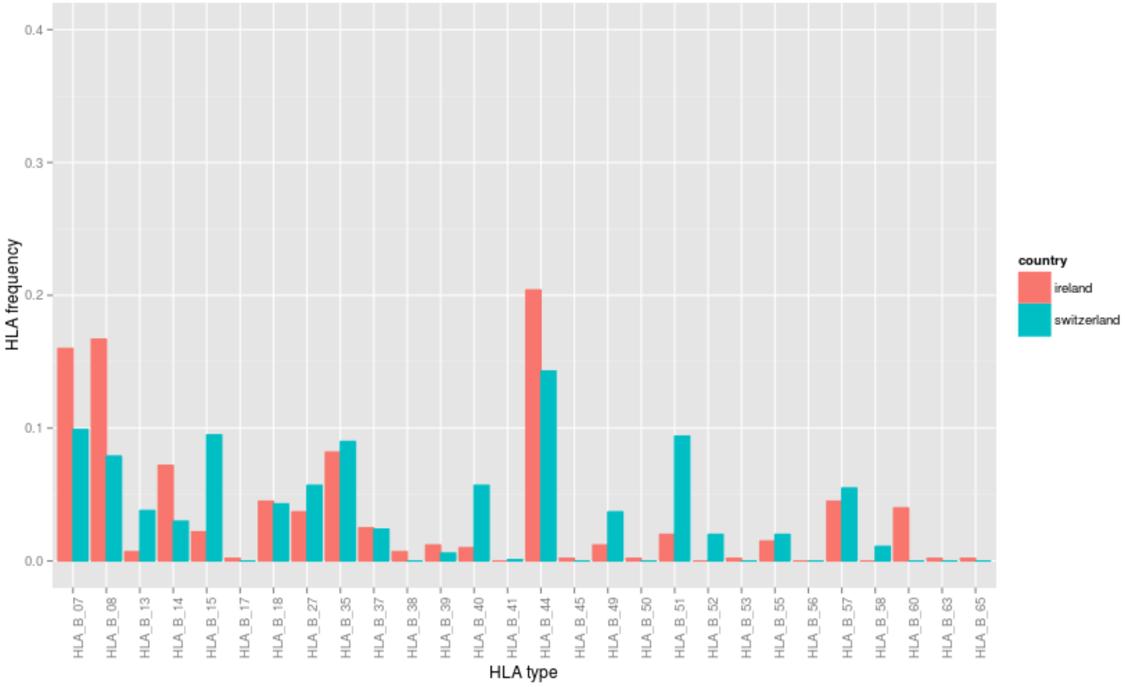


Supplementary Figure 5. HLA B subype diversity between the two populations.

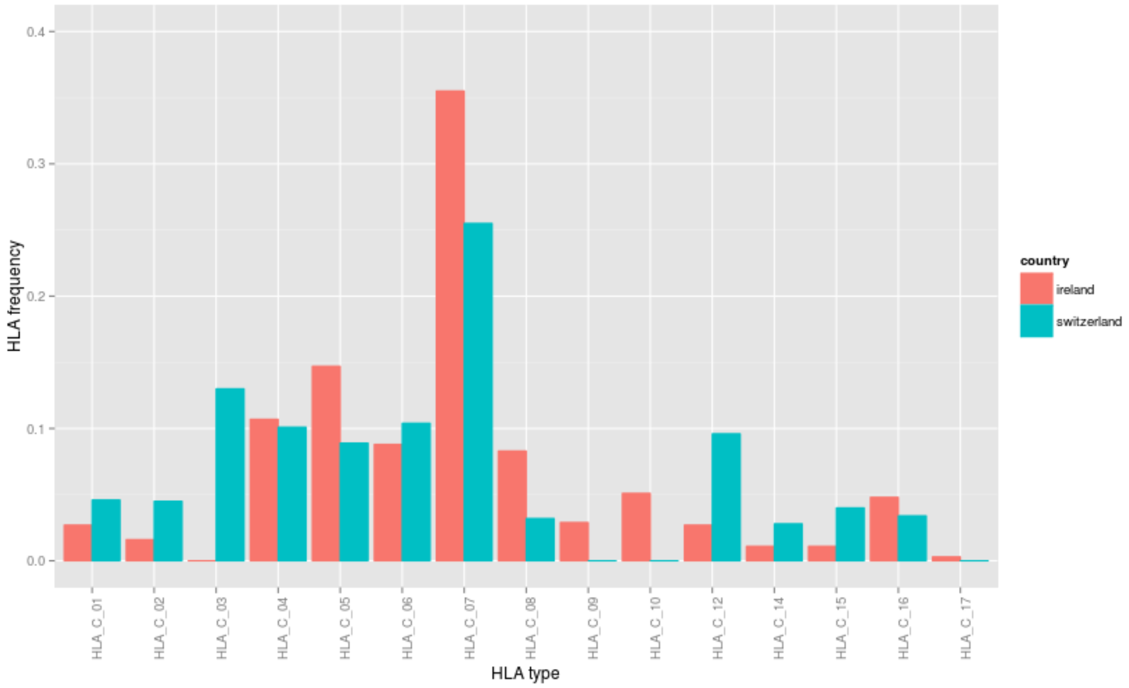


Supplementary Figure 6. HLA C diversity between the two populations.

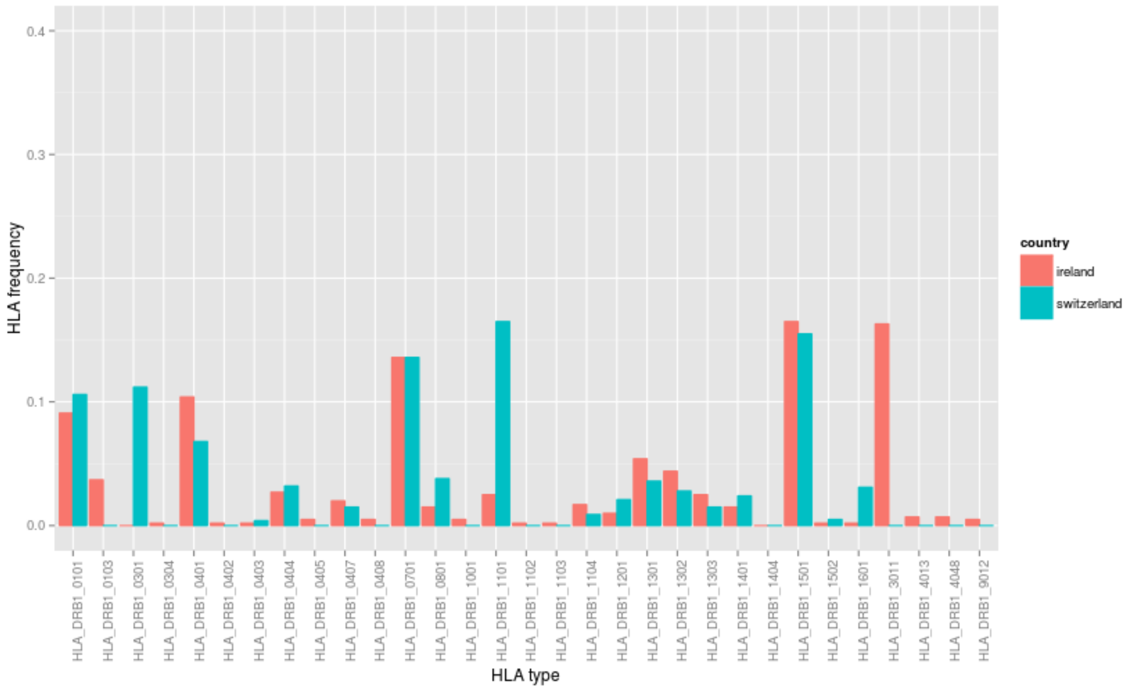


Supplementary Figure 7. HLA-DRB1 diversity between the two populations.

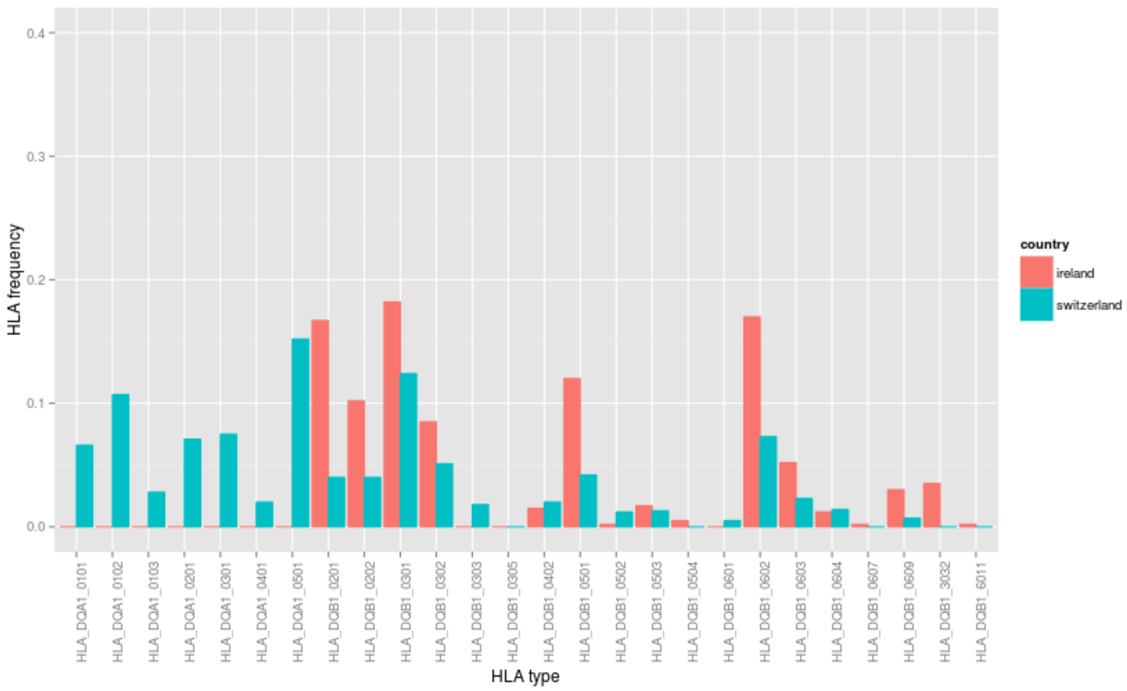


Supplementary Figure 8. HLA DRQ subtype distribution between the two populations.

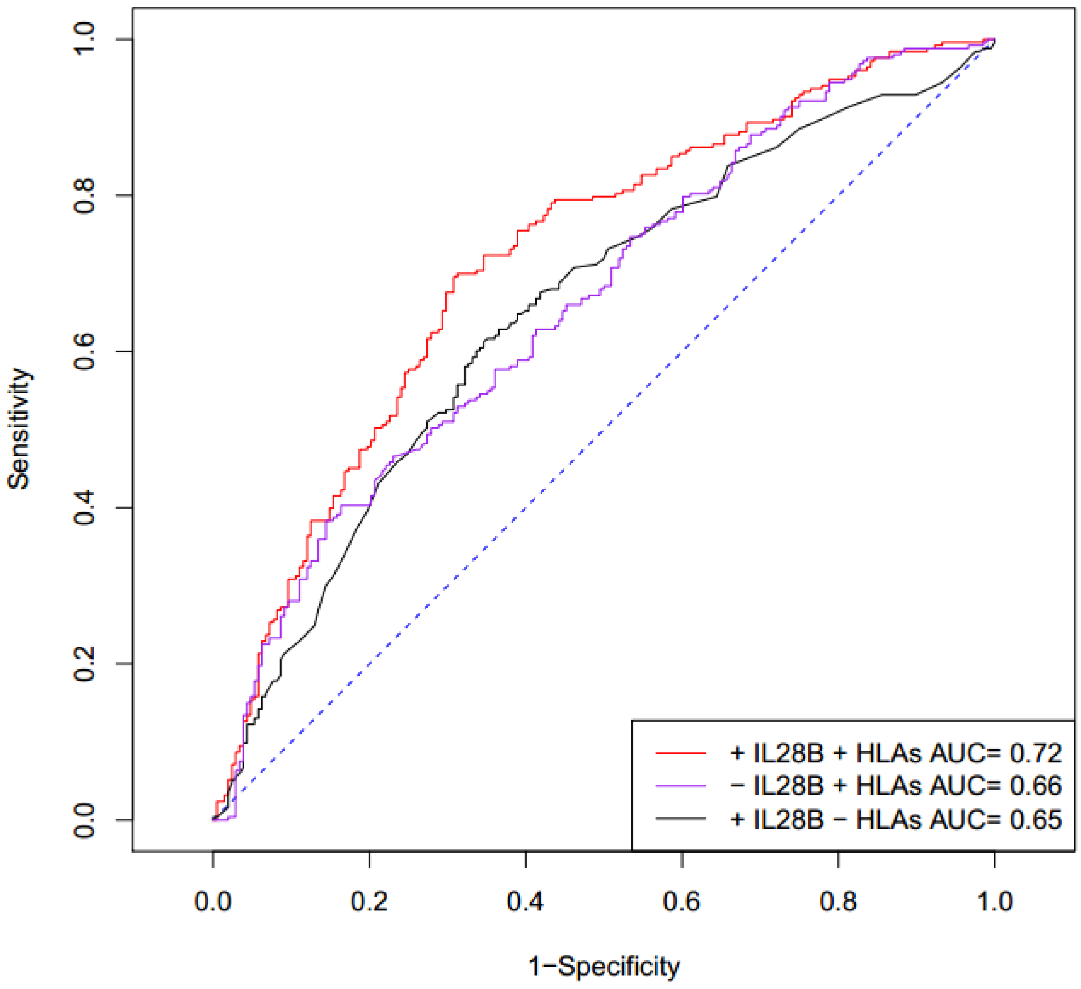


Supplementary Figure 9. ROC curve illustrating the performance of different regression models with and without the class I and class II MHC and *IFNL3* (*IL28B*) status within Swiss cohort

Supplementary Table 1. Patient demographics of the Swiss HIV cohort

| Demographic | |
|---|---|
| Sex | Female 178 (38%); Male 283 (61%) |
| Ethnicity | White 444 (96%)<br>Black 7 (1.5%)<br>Asian 3 (<1%)<br>Hispano-American 3 (<1%)<br>Other 4 (<1%) |
| Age | Minimum:- 18 years<br>1st Quartile 27 years<br>Median 32 years<br>Mean 32.34 years<br>3rd Quartile 37 years<br>Maximum:- 73 years |
| HCV genotype | Unknown: 219<br>1 8 (3.3%)<br>1A 64 (26.4%)<br>1B 40 (16.2%)<br>2A/2C 5 (2.06%)<br>2B 3 (1.23%)<br>3 1 (0.41%)<br>3A 77 (31.8%)<br>4 10 (4.13%)<br>4A 8 (3.3%)<br>4A/4C 1 (0.41%)<br>4C/4D 19 (7.85%)<br>4F 1 (0.41%) |
| HIV coinfected | 100% |

Supplementary Table 2. HLA Class I associations with viral outcome within the Irish cohort, Fishers exact test (n=319, PCR positive n=196, PCR negative n=123, spontaneous resolvers), listed in order of significant p values

| HLA | Chronic n=196 | Resolved n=123 | P value | Odds Ratio (95% CI) |
|---|---|---|---|---|
| A*03 | 43 | 44 | 0.01 | 0.5 (0.3 to 0.9) |
| B*27 | 8 | 14 | 0.021 | 0.3 (0.1 to 0.9) |
| Cw*12 | 9 | 14 | 0.027 | 0.4 (0.1 to 1) |
| Cw*01 | 5 | 10 | 0.029 | 0.3 (0.08 to 1) |
| B*15 | 6 | 11 | 0.037 | 0.3 (0.1 to 1) |
| Cw*04 | 42 | 15 | 0.037 | 2 (1 to 4) |
| B*55 | 9 | 1 | 0.1 | 5.8 (0.8 to 259) |
| Cw*03 | 11 | 13 | 0.127 | 0.5 (.2 to 1.3) |
| B*08 | 66 | 31 | 0.133 | 1.5 (0.9 to 2.6) |
| A*30 | 7 | 1 | 0.159 | 4.5 (0.6 to 205) |
| B*07 | 50 | 41 | 0.161 | 0.7 (0.4 to 1.2) |
| B*51 | 12 | 3 | 0.176 | 2.6 (0.7 to 15) |
| B*35 | 33 | 14 | 0.198 | 1.6 (0.8 to 3.3) |
| Cw*09 | 9 | 2 | 0.2 | 3 (0.6 to 28) |
| A*31 | 5 | 6 | 0.35 | 0.5 (0.1 to 2) |
| A*02 | 97 | 55 | 0.4 | 1.2 (0.8 to 2) |
| A*23 | 11 | 4 | 0.4 | 1.8 (0.5 to 7.8) |
| B*49 | 3 | 4 | 0.4 | 0.5 (0.1 to 2.8) |
| Cw*10 | 10 | 9 | 0.47 | 0.7 (0.2 to 2) |
| A*32 | 16 | 7 | 0.5 | 1.5 (0.5 to 4.4) |
| A*25 | 10 | 8 | 0.63 | 0.8 (0.3 to 2.3) |
| B*40 | 10 | 8 | 0.63 | 0.8 (0.3 to 2.3) |
| A*11 | 34 | 18 | 0.64 | 1.2 (0.6 to 2.4) |
| Cw*14 | 4 | 1 | 0.65 | 2.5 (0.3 to 126) |
| B*57 | 13 | 10 | 0.66 | 0.8 (0.3 to 2.1) |
| B*38 | 3 | 3 | 0.68 | 0.6 (0.1 to 4.7) |

| | | | | |
|---|---|---|---|---|
| A*24 | 23 | 12 | 0.71 | 1.3  (0.6 to 2.8) |
| B*14 | 26 | 14 | 0.73 | 1.2 (0.6 to 2.6) |
| A*26 | 5 | 4 | 0.74 | 0.8 (0.2 to 4) |
| B*37 | 9 | 7 | 0.79 | 0.8 (0.3 to 2.6) |
| A*01 | 80 | 48 | 0.82 | 1.1 (0.7 to 1.8) |
| B*18 | 18 | 10 | 0.84 | 1.1 (0.5 to 2.9) |
| Cw*08 | 26 | 15 | 0.86 | 1.1 (0.5 to 2.3) |
| Cw*05 | 53 | 32 | 0.9 | 1.1 (0.6 to 1.8) |
| Cw*07 | 122 | 75 | 0.91 | 1.1 (0.6 to 1.7) |
| A*29 | 23 | 15 | 1 | 1 (0.5 to 2.1) |
| A*68 | 12 | 8 | 1 | 1 (0.3 to 2.7) |
| B*13 | 3 | 1 | 1 | 1.9 (0.2 to 100) |
| B*39 | 4 | 3 | 1 | 0.8 (0.1 to 5.8) |
| B*44 | 77 | 49 | 1 | 1 (0.6 to 1.6) |
| B*60 | 10 | 6 | 1 | 1 (0.3 to 3.6) |
| Cw*02 | 7 | 5 | 1 | 0.9 (0.2 to 3.6) |
| Cw*06 | 30 | 19 | 1 | 1 (0.5 to 2) |
| Cw*15 | 4 | 2 | 1 | 1.3 (0.2 to 14) |
| Cw*16 | 18 | 11 | 1 | 1 (0.4 to 2.5) |
| Cw*17 | 1 | 1 | 1 | 0.6 (0.008 to 50) |

Supplementary Table 3. HLA Class I associations with viral outcome in Irish patients who carry the favorable IL28B CC genotype (Fishers exact test used to generate p values), listed in order of significant p values

| HLA | Chronic n=62 | Resolved n=94 | P value | Odds Ratio (95% CI) |
|---|---|---|---|---|
| A*03 | 10 | 34 | 0.007 | 0.34 (0.14 to 0.8) |
| Cw*16 | 11 | 6 | 0.035 | 3.1 (1 to 11) |
| Cw*09 | 5 | 1 | 0.037 | 8.1 (0.9 to 389) |
| Cw*03 | 1 | 10 | 0.05 | 0.14 (0.003 to 1) |
| A*11 | 15 | 12 | 0.09 | 2.2 (0.9 to 5.6) |
| Cw*12 | 3 | 12 | 0.164 | 0.35 (0.06 to 1.4) |
| B*07 | 15 | 32 | 0.2 | 0.62 (0.3 to 1.3) |
| B*51 | 4 | 2 | 0.2 | 3.1 (0.4 to 36) |
| B*35 | 12 | 11 | 0.25 | 1.8 (0.7 to 5) |
| A*02 | 34 | 42 | 0.25 | 1.5 (0.8 to 3) |
| Cw*04 | 13 | 13 | 0.28 | 1.6 (0.6 to 4.2) |
| B*55 | 3 | 1 | 0.3 | 4.7 (0.4 to 251) |
| A*68 | 2 | 7 | 0.3 | 0.4 (0.04 to 2.3) |
| B*40 | 2 | 7 | 0.3 | 0.4 (0.04 to 2.3) |
| A*29 | 10 | 10 | 0.34 | 1.6 (0.6 to 4.6) |
| Cw*02 | 4 | 3 | 0.44 | 2.1 (0.3 to 14.7) |
| B*08 | 20 | 25 | 0.47 | 1.3 (0.6 to 2.8) |
| B*57 | 3 | 8 | 0.53 | 0.5 (0.09 to 2.4) |
| Cw*07 | 37 | 61 | 0.6 | 0.8 (0.4 to 1.6) |
| B*44 | 25 | 34 | 0.6 | 1.2 (0.6 to 2.4) |
| B*14 | 7 | 14 | 0.6 | 0.7 (0.2 to 2.1) |
| Cw*08 | 7 | 14 | 0.6 | 0.7 (0.2 to 2.1) |
| A*26 | 1 | 4 | 0.65 | 0.4 (0.007 to 3.9) |
| B*49 | 1 | 4 | 0.65 | 0.4 (0.007 to 3.9) |
| A*31 | 2 | 5 | 0.7 | 0.6 (0.06 to 3.8) |
| A*32 | 2 | 5 | 0.7 | 0.6 (0.06 to 3.8) |

| | | | | |
|---|---|---|---|---|
| B*37 | 2 | 5 | 0.7 | 0.6 (0.06 to 3.8) |
| Cw*01 | 2 | 5 | 0.7 | 0.6 (0.06 to 3.8) |
| Cw*05 | 14 | 24 | 0.7 | 0.9 (0.4 to 1.9) |
| B*60 | 4 | 4 | 0.71 | 1.5 (0.3 to 8.7) |
| A*01 | 23 | 38 | 0.74 | 0.9 (0.4 to 1.8) |
| B*15 | 3 | 7 | 0.74 | 0.6 (0.1 to 3) |
| A*25 | 4 | 8 | 0.77 | 0.7 (0.2 to 3) |
| B*27 | 4 | 8 | 0.77 | 0.7 (0.2 to 3) |
| A*24 | 6 | 7 | 0.8 | 1.3 (0.4 to 4.9) |
| B*18 | 7 | 9 | 0.8 | 1.2 (0.4 to 3.9) |
| Cw*06 | 8 | 14 | 0.81 | 0.9 (0.3 to 2.3) |
| A*23 | 2 | 4 | 1 | 0.8 (0.07 to 5.4) |
| B*39 | 2 | 3 | 1 | 1 (0.08 to 9.1) |
| Cw*10 | 4 | 7 | 1 | 0.9 (0.2 to 3.5) |
| Cw*15 | 1 | 2 | 1 | 0.8 (0.01 to 15) |

Supplementary Table 4. HLA Class I associations with viral outcome in Irish cohort patients who carry the unfavorable IL28B CT/TT genotypes (Fishers exact test used to generate p values), listed in order of significant p values

| HLA | Chronic n=134 | Resolved n=29 | P value | Odds Ratio (95% CI) |
|---|---|---|---|---|
| B*27 | 4 | 6 | 0.002 | 0.12 (0.02 to 0.6) |
| Cw*01 | 3 | 5 | 0.005 | 0.11 (0.02 to 0.6) |
| B*15 | 3 | 4 | 0.02 | 0.2 (0.02 to 0.9) |
| Cw*16 | 7 | 5 | 0.04 | 0.3 (0.07 to 1.2) |
| Cw*04 | 29 | 2 | 0.07 | 3.7 (0.8 to 34) |
| Cw*07 | 85 | 14 | 0.15 | 1.9 (0.8 to 4.5) |
| B*08 | 46 | 6 | 0.2 | 2 (0.7 to 6.4) |
| Cw*08 | 19 | 1 | 0.21 | 4.6 (0.7 to 199) |
| Cw*02 | 3 | 2 | 0.22 | 0.3 (0.03 to 3.9) |
| B*44 | 52 | 15 | 0.22 | 0.6 (0.2 to 1.4) |
| A*29 | 13 | 5 | 0.32 | 0.5 (0.2 to 2) |
| Cw*17 | 1 | 1 | 0.32 | 0.2 (0.003 to 17) |
| A*03 | 33 | 10 | 0.35 | 0.6 (0.2 to 1.7) |
| A*11 | 19 | 6 | 0.4 | 0.6 (0.2 to 2.2) |
| B*13 | 2 | 1 | 0.45 | 0.4 (0.02 to 26) |
| A*01 | 57 | 10 | 0.53 | 1.4 (0.6 to 3.7) |
| A*31 | 3 | 1 | 0.55 | 0.6 (0.05 to 35) |
| B*38 | 3 | 1 | 0.55 | 0.6 (0.05 to 35) |
| Cw*14 | 3 | 1 | 0.55 | 0.6 (0.05 to 35) |
| A*24 | 17 | 5 | 0.55 | 0.7 (0.2 to 2.7) |
| B*35 | 21 | 3 | 0.6 | 1.6 (0.4 to 9) |
| B*60 | 6 | 2 | 0.63 | 0.6 (0.1 to 6.8) |
| Cw*10 | 6 | 2 | 0.63 | 0.6 (0.1 to 6.8) |
| Cw*12 | 6 | 2 | 0.63 | 0.6 (0.1 to 6.8) |
| B*07 | 35 | 9 | 0.65 | 0.8 (0.3 to 2.2) |
| B*37 | 7 | 2 | 0.66 | 0.75 (0.1 to 7.7) |

| | | | | |
|---|---|---|---|---|
| A*68 | 10 | 1 | 0.7 | 2.2 (0.3 to 102) |
| B*18 | 11 | 1 | 0.7 | 2.5 (0.3 to 112) |
| Cw*03 | 10 | 3 | 0.7 | 0.7 (0.2 to 4.2) |
| A*32 | 14 | 2 | 0.74 | 1.6 (0.3 to 15) |
| A*02 | 63 | 13 | 1 | 1.1 (0.5 to 2.7) |
| A*30 | 4 | 1 | 1 | 0.9 (0.08 to 44) |
| B*40 | 8 | 1 | 1 | 1.7 (0.2 to 82) |
| B*51 | 8 | 1 | 1 | 1.7 (0.2 to 82) |
| B*57 | 10 | 2 | 1 | 1.1 (0.2 to 11) |
| Cw*05 | 39 | 8 | 1 | 1.1 (0.4 to 3.5) |
| Cw*06 | 22 | 5 | 1 | 1 (0.3 to 3.5) |
| Cw*09 | 4 | 1 | 1 | 0.9 (0.08 to 44) |

Supplementary Table 5. HLA Class II associations with viral outcome from patients within the Irish cohort, Fishers exact test, listed in order of significant p values, n=213

| HLA | Chronic<br>n=136 | Resolved n=77 | P value | Odds Ratio (95% CI) |
|---|---|---|---|---|
| DQ*0201 | 56 | 11 | 0 | 4.2 (2 to 10) |
| DQ*0501 | 19 | 29 | 0 | 0.3 (0.1 to 0.6) |
| DR*0101 | 11 | 26 | 0 | 0.2 (0.1 to 0.4) |
| DR*3011 | 55 | 11 | 0 | 4 (2 to 9.3) |
| DR*1301 | 20 | 2 | 0.004 | 6.4 (1.5 to 58) |
| DR*0603 | 19 | 2 | 0.007 | 6.1 (1.4 to 55) |
| DQ*0202 | 33 | 8 | 0.018 | 2.8 (1.2 to 7.3) |
| DR*0401 | 20 | 22 | 0.019 | 0.4 (0.2 to 1) |
| DQ*3032 | 5 | 9 | 0.04 | 0.3 (0.1 to 1) |
| DR*1501 | 37 | 30 | 0.09 | 0.6 (0.3 to 1.1) |
| DR*0404 | 4 | 7 | 0.1 | 0.3 (0.06 to 1.3) |
| DQ*0602 | 38 | 30 | 0.13 | 0.6 (0.3 to 1.1) |
| DR*0701 | 39 | 16 | 0.25 | 1.5 (0.8 to 3.2) |
| DR*0407 | 7 | 1 | 0.26 | 4.1 (0.5 to 188) |
| DQ*0301 | 43 | 30 | 0.3 | 0.7 (0.4 to 1.4) |
| DQ*0302 | 19 | 15 | 0.33 | 0.7 (0.3 to 1.5) |
| DR*1101 | 8 | 2 | 0.33 | 2.3 (0.4 to 23) |
| DQ*0402 | 5 | 1 | 0.4 | 2.9 (0.3 to 139) |
| DR*0801 | 5 | 1 | 0.4 | 2.9 (0.3 to 139) |
| DR*1401 | 5 | 1 | 0.4 | 2.9 (0.3 to 139) |
| DQ*0503 | 6 | 1 | 0.4 | 3.5 (0.4 to 163) |
| DQ*0604 | 4 | 1 | 0.7 | 2.3 (0.2 to 115) |
| DR*1104 | 4 | 3 | 0.7 | 0.8 (0.1 to 5.3) |
| DR*1303 | 6 | 4 | 0.7 | 0.8 (0.2 to 4.2) |
| DQ*0609 | 7 | 5 | 0.8 | 0.8 (0.2 to 3.20) |
| DQ*0504 | 1 | 1 | 1 | 0.6 (0.007 to 45) |

| | | | | |
|---|---|---|---|---|
| DR*0103 | 10 | 5 | 1 | 1.1 (0.3 to 4.4) |
| DR*0405 | 1 | 1 | 1 | 0.6 (0.007 to 45) |
| DR*0408 | 1 | 1 | 1 | 0.6 (0.007 to 45) |
| DR*1001 | 1 | 1 | 1 | 0.6 (0.007 to 45) |
| DR*1201 | 3 | 1 | 1 | 1.7 (0.1 to 91) |
| DR*1302 | 12 | 6 | 1 | 1.1 (0.4 to 3.9) |
| DR*9012 | 1 | 1 | 1 | 0.6 (0.007 to 45) |

Supplementary Table 6. HLA Class II associations with viral outcome in patients from the Irish cohort who carry the favorable IL28B CC genotype (Fishers exact test used to generate p values), listed in order of significant p values

| HLA | Chronic<br>n=47 | Resolved n=56 | P value | Odds Ratio<br>(95% CI) |
|---|---|---|---|---|
| DR*0101 | 3 | 17 | 0.002 | 0.16 (0.03 to 0.6) |
| DQ*0202 | 14 | 4 | 0.004 | 5.4 (1.5 to 25) |
| DQ*0201 | 18 | 9 | 0.014 | 3.2 (1.2 to 9.3) |
| DR*03011 | 17 | 9 | 0.024 | 2.9 (1.1 to 8.5) |
| DQ*0501 | 8 | 20 | 0.045 | 0.4 (0.1 to 1) |
| DR*0701 | 17 | 11 | 0.076 | 2.3 (0.9 to 6.3) |
| DQ*0301 | 12 | 24 | 0.1 | 0.5 (0.2 to 1.1) |
| DR*0407 | 4 | 1 | 0.18 | 5 (0.5 to 256) |
| DR*3032 | 2 | 7 | 0.18 | 0.3 (0.03 to 1.8) |
| DR*1501 | 13 | 23 | 0.2 | 0.6 (0.2 to 1.4) |
| DR*0401 | 9 | 17 | 0.26 | 0.5 (0.2 to 1.5) |
| DQ*0602 | 14 | 23 | 0.3 | 0.6 (0.2 to 1.5) |
| DR*0103 | 7 | 4 | 0.3 | 2.3 (0.5 to 11.3) |
| DQ*0302 | 8 | 13 | 0.47 | 0.7 (0.2 to 2) |
| DR*1303 | 1 | 3 | 0.6 | 0.4 (0.007 to 5) |
| DR*1302 | 3 | 5 | 0.73 | 0.7 (0.1 to 3.8) |
| DQ*0609 | 3 | 4 | 1 | 0.9 (0.1 to 5.6) |
| DR*1101 | 1 | 1 | 1 | 1.2 (0.02 to 95) |
| DR*1301 | 9 | 0 | 0 | 0 |
| DQ*0603 | 9 | 0 | 0 | 0 |

Supplementary Table 7. HLA Class II associations with viral outcome in patients from the Irish cohort who carry the unfavorable IL28B CT/TT genotypes (Fishers exact test used to generate p values), listed in order of significant p values

| HLA | Chronic n=89 | Resolved n=21 | P value | Odds Ratio (95% CI) |
|---|---|---|---|---|
| DR*0101 | 8 | 9 | 0.001 | 0.14 (0.04 to 0.5) |
| DQ*0501 | 11 | 9 | 0.003 | 0.2 (0.06 to 0.6) |
| DQ*0201 | 38 | 2 | 0.005 | 7 (1.5 to 66) |
| DR*3011 | 38 | 2 | 0.005 | 7 (1.5 to 66) |
| DR*0401 | 11 | 5 | 0.18 | 0.5 (0.1 to 2) |
| DQ*03032 | 3 | 2 | 0.24 | 0.3 (0.04 to 4.3) |
| DR*1001 | 1 | 1 | 0.35 | 0.2 (0.003 to 19) |
| DR*0103 | 3 | 1 | 0.58 | 0.7 (0.05 to 38) |
| DQ*0602 | 24 | 7 | 0.6 | 0.7 (0.2 to 2.4) |
| DR*1501 | 24 | 7 | 0.6 | 0.7 (0.2 to 2.4) |
| DR*1302 | 9 | 1 | 0.7 | 2.2 (0.3 to 103) |
| DQ*0301 | 31 | 6 | 0.8 | 1.3 (0.4 to 4.6) |
| DQ*0202 | 19 | 4 | 1 | 1.2 (0.3 to 5.3) |
| DQ*0302 | 11 | 2 | 1 | 1.3 (0.3 to 13.4) |
| DQ*0503 | 6 | 1 | 1 | 1.4 (0.2 to 70) |
| DQ*0603 | 10 | 2 | 1 | 1.2 (0.2 to 12.2) |
| DQ*0609 | 4 | 1 | 1 | 1 (0.1 to 49) |
| DR*0701 | 22 | 5 | 1 | 1 (0.3 to 4.1) |
| DR*1101 | 7 | 1 | 1 | 1.7 (0.2 to 81) |
| DR*1301 | 11 | 2 | 1 | 1.3 (0.3 to 13.4) |
| DR*1303 | 5 | 1 | 1 | 1.2 (0.1 to 59) |
| DR*1401 | 5 | 1 | 1 | 1.2 (0.1 to 59) |

Supplementary Table 8. Univariate Logistic Regression analysis within Swiss cohort showing results from HLAs that have a p-value<=0.3 and where the number of patients with the HLA within the cohort is >=5.

| HLA | Number patients | OR(CI) | P-value |
|---|---|---|---|
| HLA_A_11 | 47 | 2.43 (1.32-4.83) | 0.01 |
| HLA_DQB1_0302 | 62 | 1.81 (1.09-3.08) | 0.02 |
| HLA_B_35 | 66 | 1.74(1.07-2.92) | 0.03 |
| HLA_C_14 | 24 | 0.4(0.16-0.93) | 0.04 |
| HLA_C_16 | 29 | 2.34(1.05-5.79) | 0.05 |
| HLA_C_07 | 203 | 0.74(0.54-1.01) | 0.06 |
| HLA_DQB1_0301 | 147 | 0.73(0.52-1.03) | 0.07 |
| HLA_C_04 | 84 | 1.53(0.97-2.46) | 0.07 |
| HLA_A_23 | 19 | 2.38(0.91-7.2) | 0.09 |
| HLA_B_08 | 63 | 0.65(0.38-1.1 | 0.11 |
| HLA_DRB1_0801 | 32 | 0.56(0.26-1.15) | 0.12 |
| HLA_DQA1_0401 | 32 | 0.56(0.26-1.15) | 0.12 |
| HLA_DQB1_0402 | 32 | 0.56(0.26-1.15) | 0.12 |
| HLA_A_02 | 223 | 0.8(0.61-1.06) | 0.12 |
| HLA_DRB1_0401 | 37 | 1.62(0.87-3.08) | 0.13 |
| HLA_DRB1_1101 | 110 | 0.74(0.5-1.1) | 0.14 |
| HLA_A_29 | 32 | 1.69(0.83-3.66) | 0.16 |
| HLA_A_24 | 58 | 0.69(0.4-1.19) | 0.18 |
| HLA_B_40 | 46 | 0.68(0.36-1.25) | 0.21 |
| HLA_B_49 | 30 | 0.67(0.33-1.32) | 0.25 |
| HLA_C_05 | 75 | 1.33(0.82-2.18) | 0.25 |
| HLA_C_15 | 32 | 1.45(0.75-2.97) | 0.28 |
| HLA_DQA1_0103 | 38 | 1.43(0.76-2.79) | 0.28 |
| HLA_DRB1_1201 | 18 | 0.62(0.24-1.5) | 0.29 |
